# Identification of a Four Hypoxia-Associated Long Non-Coding RNA Signature and Establishment of a Nomogram Predicting Prognosis of Clear Cell Renal Cell Carcinoma

**DOI:** 10.3389/fonc.2021.713346

**Published:** 2021-07-27

**Authors:** Hualin Chen, Yang Pan, Xiaoxiang Jin, Gang Chen

**Affiliations:** Department of Urology, The First Affiliated Hospital of Chongqing Medical University, Chongqing, China

**Keywords:** clear cell renal cell carcinoma, hypoxia, lncRNA signature, nomogram, The Cancer Genome Atlas (TCGA)

## Abstract

To identify novel hypoxia-associated long non-coding RNAs (lncRNAs) as potential biomarkers, we developed a risk stratification signature and constructed a prognosis prediction nomogram of clear cell renal cell carcinoma (ccRCC). Hypoxia-related lncRNAs were identified through Pearson correlation analysis between the expression profiles of hypoxia-related differentially expressed genes and lncRNAs from The Cancer Genome Atlas Kidney Renal Clear Cell Carcinoma (TCGA-KIRC) dataset. Then, a signature of four key lncRNAs (*COMETT*, *EMX2OS*, *AC026462.3*, and *HAGLR*) was developed. The four lncRNAs were downregulated in high-grade, advanced stage, and high-risk ccRCC. The signature had an independent and long-standing prognosis prediction ability up to a 10-year follow-up. Notably, the risk score was significantly positively correlated with the infiltration abundances of six immune cells from the Tumor IMmune Estimation Resource (TIMER). The gene set enrichment analysis (GSEA) also suggested that the signature was involved in metabolism and tumorigenesis, which were closely related to the hypoxic tumor microenvironment. Ultimately, a nomogram of signature, age, stage, and grade, was built to predict the individual long-term survival possibility. Finally, the expressions of four lncRNAs were validated by quantitative real-time PCR (qRT-PCR). Our study identified a four-lncRNA signature and established a prognostic nomogram that reliably predicts survival in ccRCC. The findings may be beneficial to therapeutic customization and medical decision-making.

## Introduction

With 208,500 new cases diagnosed worldwide each year, renal cancer is one of the most common urological malignancies and most of them are pathologically ccRCC (1). Their invasive features contribute to unfavorable prognosis, including high morbidity and mortality (2). With the advance in diagnosis and treatment, targeted therapeutic strategies have improved the overall survival rate of ccRCC to a certain degree. However, for high-grade and advanced-stage ccRCC, the survival rate is still low (3). Hence, identification of novel biomarkers and customization of therapeutic options are urgently needed to improve the prognosis of patients with ccRCC.

Due to the imbalance between vascular nutrient supply and the rate of tumor cell proliferation, hypoxia is an intrinsic feature of solid tumors (4). Many studies have uncovered the critical roles of hypoxia in the tumor microenvironment, including cell proliferation and differentiation and tumor angiogenesis and immune infiltration. Hypoxia can activate the hypoxia-inducible factors and then induce adaptive changes within a cancer cell, which results in tumor progression and treatment resistance (5). Previous studies have explored the close relations between the hypoxia microenvironment and several cancers, including gastric cancer, glioblastoma, colorectal cancer, breast cancer, and hepatocellular carcinoma (6–10). However, limited evidence is reported about the possible mechanisms of hypoxia affecting the tumorigenesis, progression, and distant metastasis of ccRCC. Therefore, further efforts on the relationship between hypoxia and the biological behaviors of ccRCC are required to develop novel treatment methods.

LncRNA refers to RNA that does not encode proteins and has a length greater than 200 nucleotides. By coordinating with other molecules, lncRNA can regulate several physiological processes, and their dysfunction may contribute to pathologies, including cancer and infectious diseases (11). One study by Che et al. uncovered the potential relationships between dysregulation of key lncRNA and ccRCC invasion, suggesting the critical roles played by lncRNA in the ccRCC microenvironment (12).

By taking advantage of high-throughput sequencing data obtained from the TCGA database and advanced bioinformatics analyses, we carried out this present study to explore lncRNAs targeting hypoxia-related genes and further constructed a hypoxia-associated prognostic signature for ccRCC patients.

## Materials and Methods

**Figure 1:**
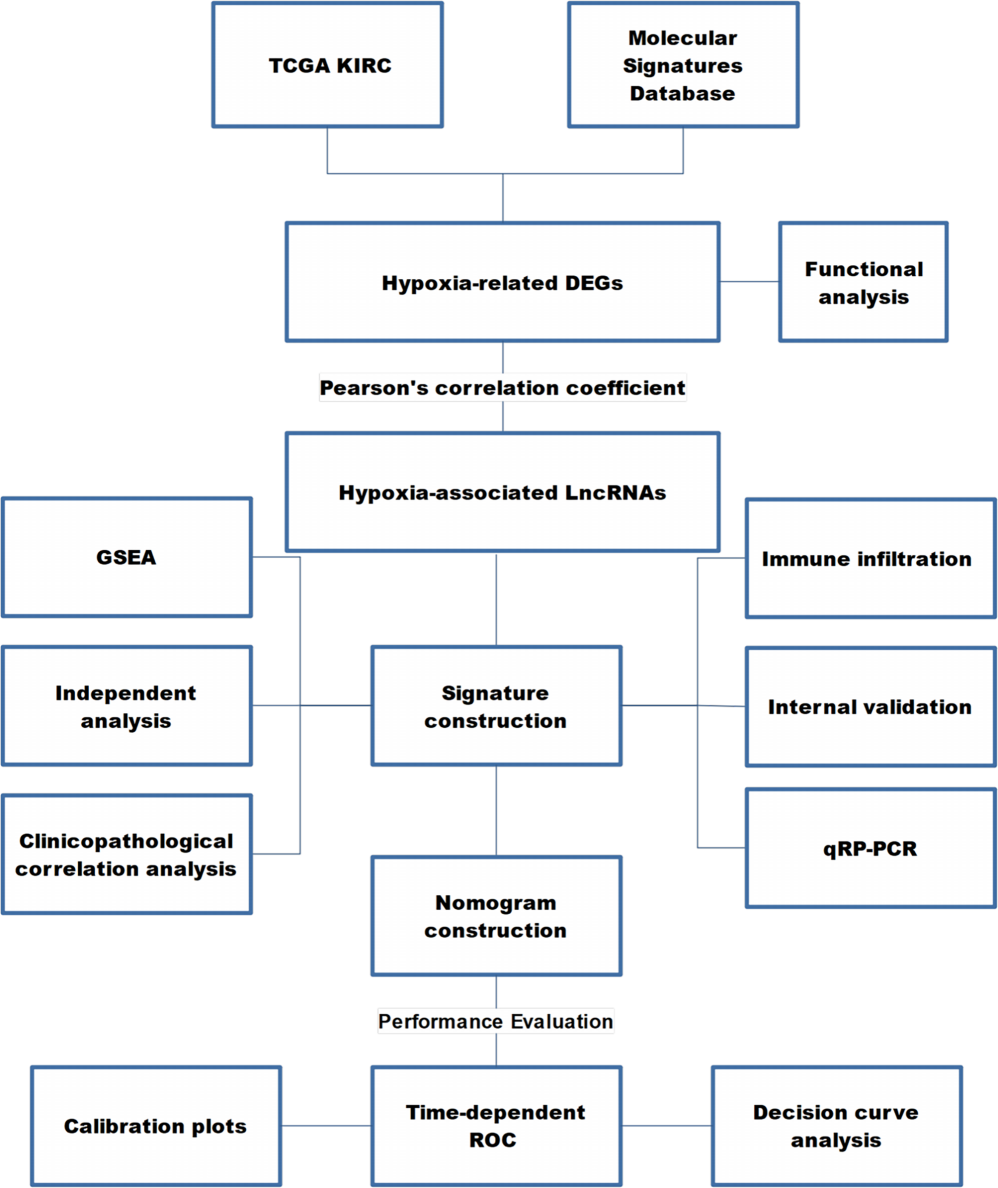
The workflow of this study. TCGA KIRC, The Cancer Genome Atlas Kidney Renal Clear Cell Carcinoma; DEG, differentially expressed gene; LncRNA, long non-coding RNA; GSEA, gene set enrichment analysis; ROC, receiver operating characteristic curve.

**Figure 1** presents the workflow of our study.

### Data Acquisition

The gene expression data of level 3 RNA-seq fragments per kilobase million (FPKM) dataset and clinical data were retrieved from TCGA KIRC (https://portal.gdc.cancer.gov/) and UCSC Xena (https://xenabrowser.net/), respectively.

Two hundred hypoxia-related genes were downloaded from the hallmark gene sets of the Molecular Signatures Database (M5891, https://www.gsea-msigdb.org/gsea/msigdb/).

### Identification of Differentially Expressed Hypoxia-Related Genes and Enrichment Analysis

The expression profiles of the hypoxia-related genes were extracted from the TCGA KIRC dataset. Then, we applied the limma R package to select the differentially expressed hypoxia-related genes between ccRCC samples and normal renal samples (13). Adjusted P-value (adj. P-value) of 0.05 and |logFC| ≥1 were set as the cut-off criteria. The online tool Metascape was used for enrichment analysis (https://metascape.org/gp/index.html) (14).

### Identification of lncRNAs Targeting Hypoxia-Related Genes

To screen out the lncRNAs targeting differentially expressed hypoxia-related genes, we used Pearson correlation analysis between the expression profiles of all lncRNAs and the differentially expressed hypoxia-related genes. |R| >0.6 and P-value <0.01 were set as the cut-off criteria.

### Construction of a Hypoxia-Associated lncRNAs Signature

The inclusion criteria for ccRCC samples in lncRNA signature construction and validation included (1): samples with complete lncRNA expression levels, and (2) samples with total survival time over 30 days. Subsequently, the eligible ccRCC samples were randomly divided into the training and testing cohorts on the ratio of 7:3 by caret R package.

First, we performed univariate Cox regression analysis in the training cohort to screen the prognostic hypoxia-associated lncRNAs by the cut-off value (P < 0.01). Then, we performed the Least Absolute Shrinkage and Selector Operation (LASSO) analysis, which can reduce the estimation variance and provide an explicable final model. Then, we used the multivariate Cox regression analysis to establish a prognostic signature. Furthermore, we calculated the risk score of each ccRCC using the formula: Risk score = ExplncRNA1 × CoeflncRNA1 + ExplncRNA2 × CoeflncRNA2 + … ExplncRNA(n)× CoeflncRNA(n). In this equation, “ExplncRNA” represented the expression level of a lncRNA, and “CoeflncRNA” was the regression coefficient of a lncRNA calculated by the multivariate Cox regression analysis. Next, the median value of the risk score was set as the threshold to divide the training cohort into high- and low-risk groups. The Kaplan–Meier (KM) survival curve by log-rank test and time-dependent receiver operating characteristic (ROC) curve were plotted to assess the signature’s predictive ability. Further, the signature’s stability and reliability were verified in the testing, and the entire cohorts using similar methods. The glmnet, survival, and survminer R packages were used for these analyses.

We then used the univariate and multivariate Cox regression analyses to assess the independent predictive ability of the signature based on the signature and clinicopathological characteristics in the three subgroups of the TCGA KIRC dataset. We also performed the stratified analysis to analyze the clinicopathological factors with significance (P < 0.05).

### Assessment of Immune Cell Infiltration and Immune Microenvironment

Published studies have proposed the close relationships between hypoxia, tumor immunity, and immune microenvironment. Hence, we performed a correlation analysis between risk score and infiltration abundances of six immune cells downloaded from the TIMER (https://cistrome.shinyapps.io/timer/) (15). Additionally, the ESTIMATE algorithm was used to assess the immune infiltration in ccRCC (16). By clusterProfiler R package, GSEA was performed on the expression matrices of high- and low-risk groups to uncover the possible mechanism of risk level affecting hypoxia and tumor microenvironment (17).

### Development and Evaluation of a Nomogram

As nomograms can quantitatively assess the prognosis of tumor patients by interpreting the prediction model into a single numerical value, they are widely used in research and clinics to predict the survival probability of patients. In our study, we developed a nomogram comprising age, stage, grade, and risk score together. The total points of the nomogram indicated the probability of 5-, 7- and 10-year overall survival of ccRCC patients.

To test the performance of the constructed nomogram, time-dependent ROC curves and calibration plots were produced. As a method for evaluating molecular markers, diagnostic tests, and clinical predictive models, the decision curve analysis (DCA) was used to assess the predictive ability of the nomogram.

### qRT-PCR Analysis

Ten pairs of ccRCC (pathologically confirmed by two independent senior pathologists of our single-center through FFPE slices) and tumor-adjacent normal tissues were collected from patients who underwent partial/radical nephrectomy from March 2021 to April 2021 from the department of urology in the First Affiliated Hospital of Chongqing Medical University. Tissues were excised and immediately transferred into liquid nitrogen. All patients were informed, and written informed consent was provided. The study was conducted according to the clinical practice guidelines of the International Conference on Harmonization and the Declaration of Helsinki. This study protocol was approved by the ethical committee of the Affiliated Hospital of Chongqing Medical University.

Total RNA from ccRCC and tumor-adjacent normal tissues was extracted using an UNIQ-10 column Total RNA Extraction Kit (Sangon Biotech). The RNA concentration and purity were assessed using a SMA4000 microspectrophotometer (Merinton Instrument, Inc) and by RNA electrophoresis with DYY-6C electrophoresis apparatus (Liuyi, Beijing). Reversed-transcription was performed using a RR047A cDNA synthesis kit (TaKaRa, China). Quantitative PCR was performed for the four lncRNAs (*COMETT*, *EMX2OS*, *AC026462.3*, and *HAGLR*) using a 2× SG Fast qPCR Master Mix (High Rox, B639273, BBI) with the Step One Plus fluorescence quantitative PCR instrument (ABI, Foster, CA, USA), GAPDH is used for internal control gene. The primers designed by Primer Premier 5.0 are listed in **Table 1**.

**Table 1:** Primer sequences.

	Primer sequences (5′–3′)
COMETT forward	ATCTGAAGGCAGTCTGCTGG
COMETT reverse	TGCGGTTTCTGAGCCCTAAG
EMX2OS forward	CCCCTATCACTACCACCCCA
EMX2OS reverse	GCACAAACAGAGGTACCCGA
AC026462.3 forward	CAGCCAAGTGTGTGAACAGC
AC026462.3 reverse	GTGGCATCCCAATCAGTCCA
HAGLR forward	GATCCCCACCTTCCCCAAAG
HAGLR reverse	TCTCCGACTGAGGTTTGCAC
GAPDH forward	TGGGTGTGAACCATGAGAAGT
GAPDH reverse	TGAGTCCTTCCACGATACCAA

## Results

### Identification of Differentially Expressed Hypoxia-Related Genes and Enrichment Analysis

**Figure 2:**
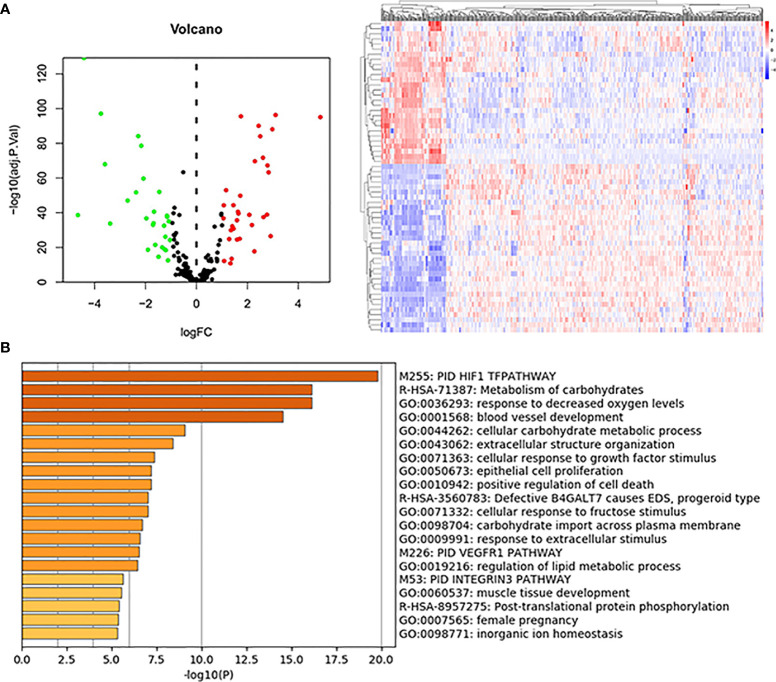
DE-hypoxia-related gene identification and enrichment analysis. **(A)** Volcano plot and heatmap of the DE-hypoxia-related genes identified from the TCGA KIRC dataset. In the volcano plot, red dots represent upregulated genes, and green dots represent downregulated ones. **(B)** Enrichment analysis of DE-hypoxia-related genes by online tool Metascape (https://metascape.org/). The top 20 enriched terms are listed, and the color of the bar represents the value of -log10(P-value).

Sixty-one differentially expressed hypoxia-related genes were obtained (**Figure 2A**) and were subjected to enrichment analysis. As listed in **Figure 2B**, PID HIF1 TFPATHWAY, metabolism of carbohydrates, response to decreased oxygen levels, blood vessel development, and extracellular structure organization were the most enriched terms, suggesting the critical roles of the hypoxia-related genes in the tumor microenvironment.

### lncRNAs Targeting Hypoxia-Related Gene Identification and Prognostic Signature Development

Eighty-seven lncRNAs, targeting sixty-one hypoxia-related genes, were identified by Pearson correlation analysis and were used for subsequent prognostic signature construction.

Under the defined criteria, 516 ccRCC samples were included and classified into a training cohort (n = 364) and a testing cohort (n = 152). We then performed univariate Cox regression analysis on the hypoxia-related lncRNAs in the training cohort and identified four lncRNAs, namely *COMETT*, *EMX2OS*, *AC026462.3*, and *HAGLR*, related to the prognosis of ccRCC (P < 0.01). Notably, the high expression of the four lncRNAs was associated with a favorable prognosis, suggesting the protective roles of these lncRNAs in patients with ccRCC.

Since the expression values of the four lncRNAs and survival data were merged, we performed the LASSO regression analysis and multivariate Cox regression analysis to develop a prognostic signature in the training cohort (**Table 2**). Based on the regression coefficients and gene expression, we calculated the risk score of each sample by the following equation: Risk score = −0.076 × Exp. *COMETT* − 0.039 × Exp. *EMX2OS* − 0.734 × Exp. *AC026462.3* − 0.065 × Exp. *HAGLR*.

**Table 2:** The four-signature lncRNAs.

LncRNA	Ensembl	Coef	HR	HR.95L	HR.95H	P-value
*COMETT*	ENSG00000231210	−0.076	0.927	0.836	1.028	0.153
*EMX2OS*	ENSG00000229847	−0.039	0.962	0.926	1.000	0.052
*AC026462.3*	ENSG00000260963	−0.734	0.480	0.260	0.888	0.019
*HAGLR*	ENSG00000224189	−0.065	0.937	0.877	1.002	0.058

Coef, regression coefficient; HR, hazard ratio; 95L-95H, 95% confidence interval of HR. P-value, calculated from multivariate Cox regression analysis.

**Figure 3:**
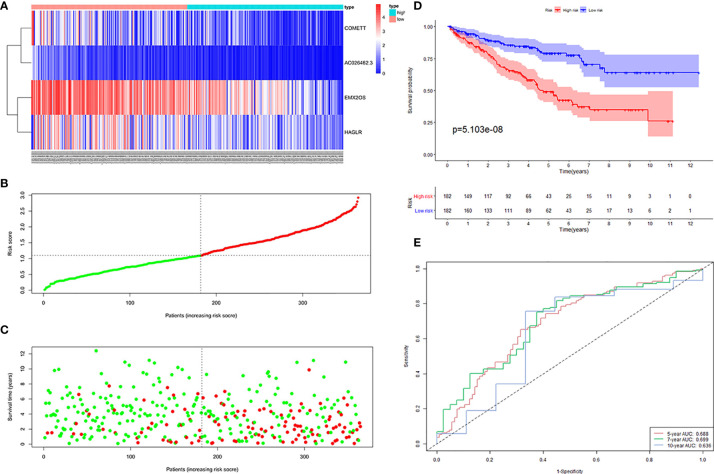
Four hypoxia-related lncRNA signature validated in the training cohort (n = 364). **(A)** Expression values of the four signature lncRNAs. The colors from red to blue correspond to the expression of a lncRNA from high to low. The risk score **(B)** and survival status **(C)** of each ccRCC patient distributed in low- and high-risk groups. **(D)** KM survival analysis between the high-risk group and low-risk group. The lower layer demonstrates the number of alive ccRCC patients in each group. **(E)** Time-dependent ROC curves of the signature.

Next, 364 samples in the training cohort were divided into high-risk and low-risk groups according to the median risk score of 1.102. The distribution of risk scores, the survival status, and the expression of the four lncRNAs for high- and low-risk ccRCC were shown in **Figures 3A–C**. To assess the prognostic value of the signature, we performed KM survival analysis and found that patients in the high-risk group had a worse prognosis than those in the low-risk group (**Figure 3D**). We then produced time-dependent ROC curves to estimate the predictive power of the constructed signature. As shown in **Figure 3E**, area under curve (AUC) for 5-, 7- and 10-year survival prediction was 0.688, 0.699, and 0.636, respectively.

### Validation of Prognostic Hypoxia-Associated lncRNAs Signature

To test the reliability and stability of the signature constructed in the training cohort, we calculated the risk score of each ccRCC in the testing and entire cohorts. We then divided the ccRCC in the two cohorts into high- and low-risk groups based on the median risk score of the training cohort.

**Figure 4:**
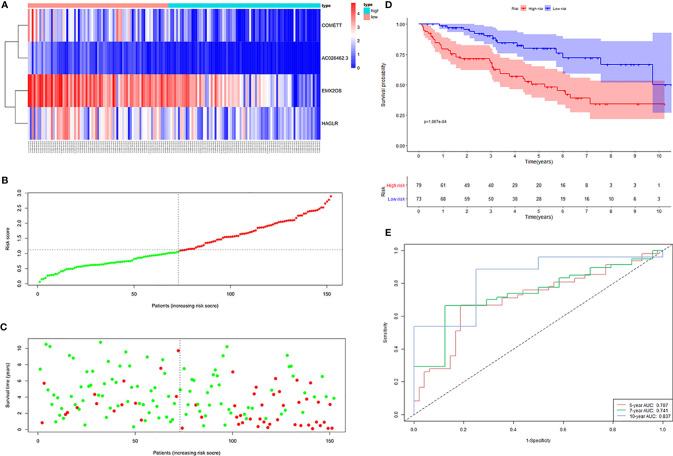
Four hypoxia-related lncRNA signature validated in the testing cohort (n = 152). **(A)** Expression values of the four signature lncRNAs. The colors from red to blue correspond to the expression of a lncRNA from high to low. The risk score **(B)** and survival status **(C)** of each ccRCC patient distributed in low- and high-risk groups. **(D)** KM survival analysis between the high-risk group and low-risk group. The lower layer demonstrates the number of alive ccRCC patients in each group. **(E)** Time-dependent ROC curves of the signature.

The distribution of risk scores, the survival status, and the expression of the four signature lncRNAs in the high- and low-risk samples of the testing cohort was shown in **Figures 4A–C**. KM survival analysis revealed that patients in the high-risk group had a worse prognosis than those in the low-risk group (**Figure 4D**). Time-dependent ROC curves showed that the AUC for 5-, 7- and 10-year survival prediction was 0.707, 0.741, and 0.837, respectively (**Figure 4E**).

**Figure 5:**
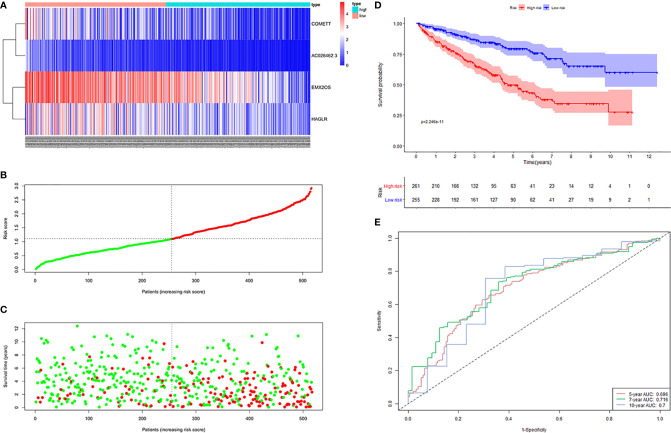
Four hypoxia-related lncRNA signature validated in entire cohort (n = 516). **(A)** Expression values of the four-signature lncRNAs. The colors from red to blue correspond to the expression of a lncRNA from high to low. The risk score **(B)** and survival status **(C)** of each ccRCC patient distributed in low- and high-risk groups. **(D)** KM survival analysis between the high-risk group and low-risk group. The lower layer demonstrates the number of alive ccRCC patients in each group. **(E)** Time-dependent ROC curves of the signature.

The distribution of risk scores, the survival status, and the expressions of the four signature lncRNAs in the high- and low-risk samples of the entire cohort was shown in **Figures 5A–C**. KM survival analysis revealed that patients in the high-risk group had a worse prognosis than those in the low-risk group (**Figure 5D**). Time-dependent ROC curves showed that the AUC for 5-, 7- and 10-year survival prediction was 0.696, 0.716, and 0.7 (**Figure 5E**).

To explore the performance of the constructed signature in progression-free survival, we performed KM analysis and generated time-dependent ROC curves. Results showed that patients with ccRCC in the high-risk group had worse PFS than those in the low-risk group. The AUC for 5-, 7- and 10-year survival prediction was 0.711, 0.703, and 0.665 (**Supplementary Figure 1**).

Generally, the four hypoxia-associated lncRNA signature presented significant prognostic value and good stability in ccRCC.

### Independent Analysis of the Signature And Clinicopathological Characteristics

Univariate Cox regression analysis and multivariate Cox regression analysis were performed on the four hypoxia-associated lncRNA signature and clinicopathological characteristics (age, gender, grade, and stage) to determine whether the prediction ability of the signature was independent of clinicopathological factors. As shown in **Table 3**, the signature showed independent prognostic prediction ability in three cohorts simultaneously. Besides, grade and stage were found to be two independent clinicopathological predictors of ccRCC.

**Table 3:** Independent prognostic analysis in three cohorts of TCGA KIRC dataset.

Cohort	Parameters	uniCox				multiCox			
		HR	HR.95L	HR.95H	P-value	HR	HR.95L	HR.95H	P-value
train	age	1.026	1.011	1.041	<0.001	1.030	1.013	1.048	<0.001
	gender	0.884	0.610	1.283	0.517	0.889	0.601	1.314	0.556
	grade	2.092	1.648	2.656	<0.001	1.321	1.007	1.732	0.044
	stage	1.866	1.590	2.191	<0.001	1.648	1.372	1.979	<0.001
	risk score	2.274	1.729	2.992	<0.001	1.606	1.191	2.165	0.002
test	age	1.031	1.005	1.058	0.019	1.029	1.000	1.059	0.051
	gender	1.171	0.647	2.118	0.602	1.235	0.678	2.251	0.490
	grade	2.731	1.821	4.095	<0.001	1.527	0.942	2.476	0.086
	stage	1.876	1.480	2.377	<0.001	1.521	1.144	2.022	0.004
	riskScore	2.388	1.601	3.562	<0.001	1.655	1.083	2.531	0.020
entire	age	1.027	1.014	1.040	<0.001	1.031	1.016	1.045	<0.001
	gender	0.961	0.702	1.316	0.806	0.981	0.710	1.354	0.907
	grade	2.254	1.836	2.767	<0.001	1.361	1.077	1.720	0.010
	stage	1.876	1.643	2.142	<0.001	1.613	1.385	1.878	<0.001
	riskScore	2.309	1.843	2.893	<0.001	1.645	1.293	2.094	<0.001

uniCox, univariate Cox regression analysis. multiCox, multivariate Cox regression analysis.

**Figure 6:**
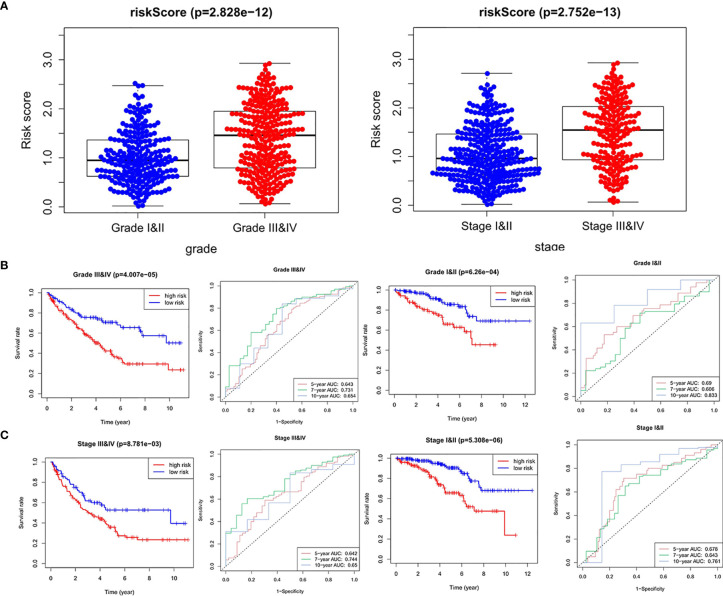
Relationships between risk score and clinicopathological factors. **(A)** Risk score between high grade (grades III and IV) and low grade (grades I and II), advanced stage (stages III and IV), and early stage (stages I and II). Stratified analysis between the high-risk group and low-risk group in the same grade **(B)** and stage **(C)**.

Next, we analyzed the relationship between the four hypoxia-associated lncRNA signature and clinicopathological characteristics. The results showed that patients with high grade (III–IV) and advanced stage (III–IV) had higher risk scores (**Figure 6A**). Stratified analysis was further carried out between the four hypoxia-associated lncRNA signature and the two clinicopathological independent predictors (grade and stage). The results of KM curves and time-dependent ROC curves showed that the four hypoxia-associated lncRNA signature had significant prognostic value and reliability for ccRCC with the same grade and stage (**Figures 6B, C** **)**. These results suggested the possible relationship between the signature and progression of ccRCC.

**Figure 7:**
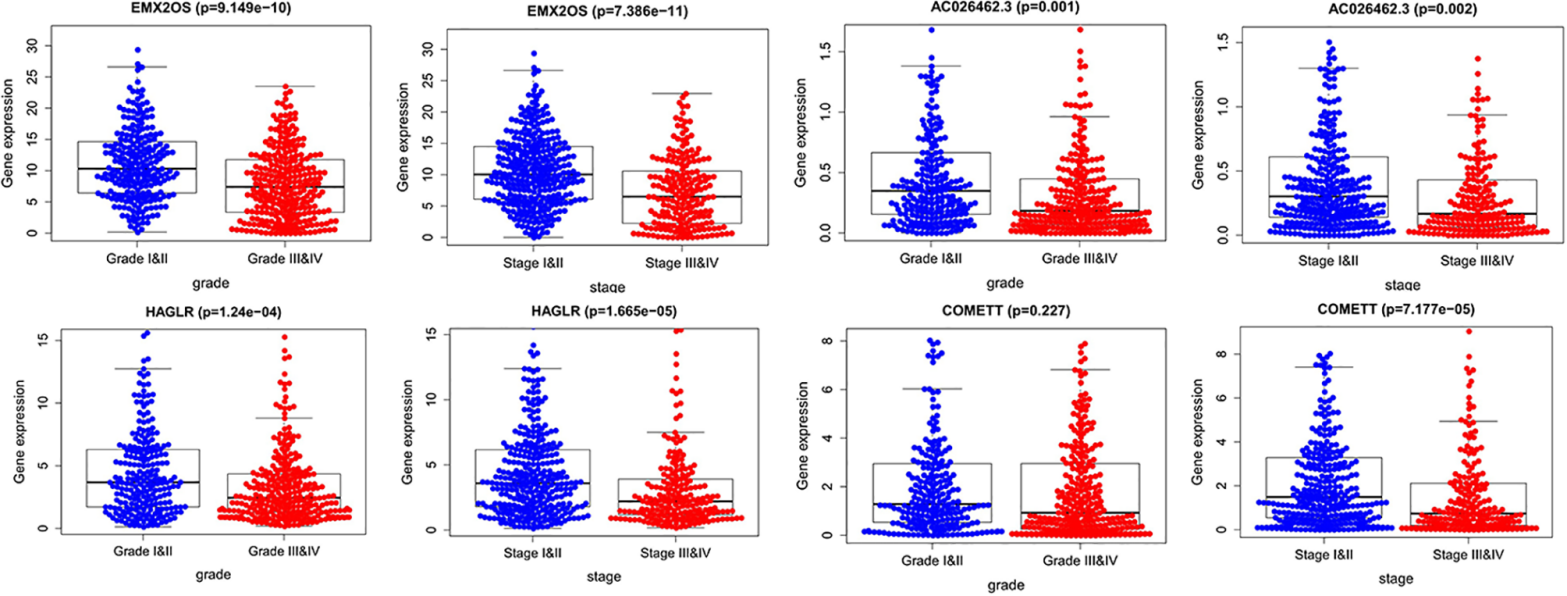
The expression of the four-signature lncRNAs between high grade and low grade, advanced stage, and early stage.

We also analyzed the relationships between expression values of the four signature lncRNAs and grade and stage. As shown in **Figure 7**, all the four lncRNAs were associated with high grade (except for *COMETT*) and advanced stage, indicating the prognostic values of the four hypoxia-associated lncRNAs.

### Correlation Analysis Between Risk Score and Infiltration Levels of Immune Cells

**Figure 8:**
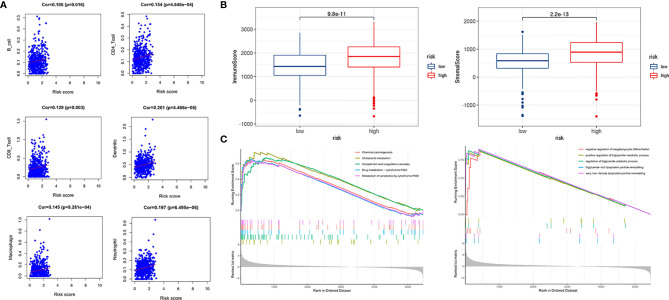
Immune infiltration correlation analysis and GSEA. **(A)** Correlation between risk score and immune infiltration abundances in ccRCC of six immune cells obtained from TIMER (https://cistrome.shinyapps.io/timer/). **(B)** Relationships between risk score and immune score, and stromal score. **(C)** GSEA in TCGA KIRC dataset.

Next, we performed a correlation analysis between the risk scores of the signature and infiltration abundances of the six immune cells obtained from TIMER. The immune cell infiltration abundances of 516 ccRCC samples were extracted from those of the whole TCGA samples. As demonstrated in **Figure 8A**, all the six immune cell types (B cells, CD4 T cells, CD8 T cells, dendritic cells, macrophages, and neutrophils) were significantly positively correlated with the risk score. Using the ESTIMATE algorithm, we obtained the stromal and immune scores of each ccRCC sample and found that the high-risk group had higher stromal and immune scores than the low-risk group (**Figure 8B**). These findings further prove the correlation between hypoxia microenvironment and tumor immune activity, providing evidence for novel tumor therapeutic strategies.

GSEA was used to conduct key gene ontology term and pathway enrichment analysis of high- and low-risk score ccRCC samples. Genes associated with high-risk score were mainly enriched in metabolism, carcinogenesis, complement, and coagulation cascades (**Figure 8C**).

### Construction of a Nomogram Based on the Four Hypoxia-Associated lncRNA Signature

**Figure 9:**
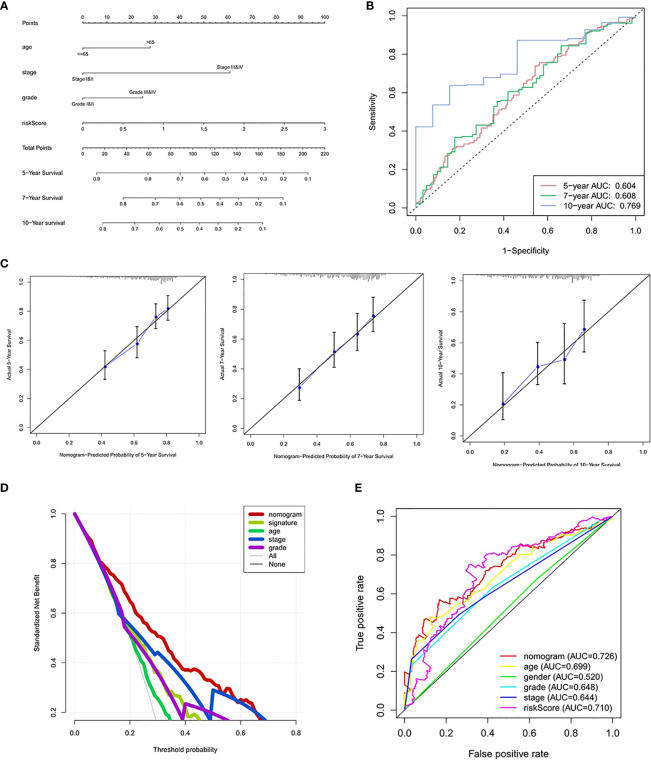
Nomogram construction and performance test. **(A)** Nomogram based on risk score, age, stage, and grade. **(B)** Time-dependent ROC curves demonstrate the performance of the nomogram in up to 10-year follow-up. **(C)** Calibration curves of the nomogram for predicting the survival outcomes at 5, 7 and 10 years. The 45-degree line represents the ideal prediction. **(D)** Decision curve analyses curve of the nomograms. **(E)** ROC curves demonstrate the performance of the nomogram, signature and clinicopathological variables in up to 10-year follow-up.

Based on the prognostic signature and clinicopathological factors, we constructed a nomogram to predict the 5-,7-, and 10-year overall survival of ccRCC (**Figure 9A**). Time-dependent ROC curves showed good discrimination of the nomogram with an AUC of 0.604, 0.608, and 0.769 at 5-, 7-, and 10-year follow-up (**Figure 9B**). Additionally, we generated the calibration plots and performed the decision curve analysis to demonstrate the good predictive effect and clinical utility of the nomogram (**Figures 9C, D** **)**. Ultimately, a multi-parameter ROC curve was generated to show the superior discrimination of the nomogram at a 10-year follow-up (**Figure 9E**).

### Validation of lncRNA Expression in Clinical Samples

**Figure 10:**
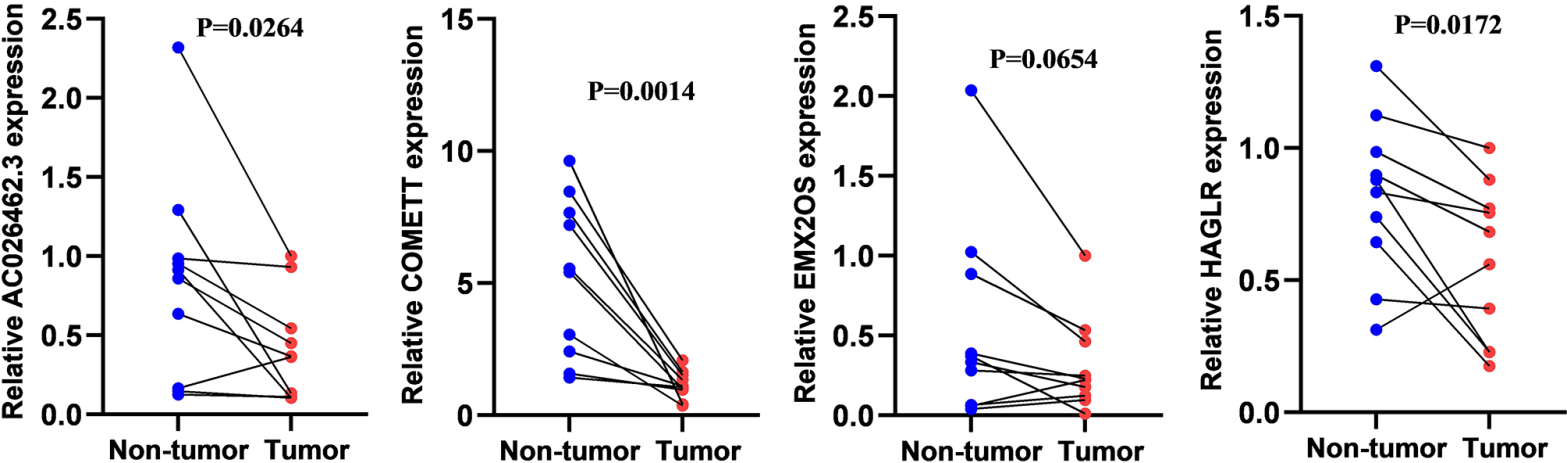
The relative expression level of *COMETT*, *EMX2OS*, *AC026462.3*, and *HAGLR* in ccRCC and tumor-adjacent normal tissues.

As demonstrated in **Figure 10**, four lncRNAs were lowly expressed in ccRCC compared to adjacent normal tissues although statistical significance was not observed in EMX2OS. The experimental results were consistent with those from the online database GEPIA (18) (http://gepia.cancer-pku.cn/index.html) (**Supplementary Figure 2**) and further supported the protective roles of the four lncRNAs in ccRCC.

## Discussion

In this study, we developed a hypoxia-associated four-lncRNA signature closely relating to the long-term prognosis of patients with ccRCC, based on the lncRNAs targeting differentially expressed hypoxia-related genes. The signature was further validated in the testing cohort and the entire cohort from the TCGA KIRC dataset. KM survival analysis in the three cohorts showed that patients in the high-risk group had poor prognosis while those in the low-risk group had favorable prognosis, indicating the good prognostic ability of the signature. Additionally, AUC for 5-, 7-, and 10-year follow-up in the three cohorts suggested stable performance in distinguishing different risk-level ccRCC. Notably, we proposed a risk score calculating formula by which we can predict the risk of patients with ccRCC once the expression values of the four lncRNAs were obtained. In the correlation analysis between signature and clinicopathological factors, we found that risk score was significantly higher in advanced stage (III–IV) and high grade stage (III–IV). Stratified analysis in the same stage and grade (I–II or III–IV) also demonstrated the good performance of the constructed signature. In general, the four-lncRNA signature demonstrated very good prognostic and discriminable ability for ccRCC.

Subsequently, the correlation between risk score and immune infiltration showed that a higher risk score was associated with high immune infiltration levels in the microenvironment of ccRCC, which was consistent with previous studies (19). GSEA enrichment analysis showed gene set in the high-risk group was mainly enriched in metabolism and tumorigenesis, suggesting the possible mechanism of hypoxia affecting the tumor microenvironment (20).

Based on this signature and other clinicopathological factors, we further constructed a nomogram to predict the up to 10-year survival probability of patients with ccRCC. More importantly, decision curve analysis further proves the superior clinical utility of the constructed nomogram to the grade and stage classification system. Based on the well-constructed nomogram, clinicians can calculate the risk score of any ccRCC patient and customize the therapeutic strategies for each patient, with an attempt to prolong the survival period.

The four signature lncRNAs, including *COMETT*, *EMX2OS*, *AC026462.3*, and *HAGLR*, were differentially expressed between ccRCC and normal samples. Downregulation of these four lncRNAs was associated with advanced stage, high grade, and poor prognosis, suggesting the protective roles in tumorigenesis, progression, and metastasis. *COMETT*, also known as *LINC01510*, has diagnostic and prognostic values in ccRCC, as reported previously (21, 22). Ma et al. also reported that low expression of *COMETT* in ccRCC was associated with poor prognosis, which was consistent with our study results. Furthermore, they found that *COMETT* acted as a tumor suppressor in ccRCC tumorigenesis by inhibiting Wnt/*β*-catenin signaling (23). The biological behaviors of *COMETT* were also studied in papillary thyroid carcinoma, colorectal cancer, and non-small cell lung cancer (24–26). *EMX2OS*, an enhancer RNA, was upregulated in low-risk ccRCC and significantly associated with low-grade, early stage, and favorable prognosis (27). Protective roles of this lncRNA were also found and validated in classical papillary thyroid cancer (28) and prostate cancer (29). The role of *EMX2OS* in hypoxia was also reported in myalgic encephalomyelitis/chronic fatigue syndrome by Yang et al. (30). High expression of *HAGLR* suggested a favorable prognosis for ccRCC, while the expression of *HAGLR* was upregulated in hepatocellular carcinoma and associated with proliferative and metastatic tumor (31). Similar findings in colon cancer and esophageal cancer were reported by Sun and Yang et al. (32, 33). Interestingly, *HAGLR* coordinating with miRNA-19a-3p/TGFBR2 could promote the healing process of a femoral neck fracture. Additionally, *HAGLR* showed strong diagnostic power for atrial fibrillation, acting as a competing endogenous RNA (34).

The main limitation of the current study was the lack of validation in an independent dataset or clinical ccRCC samples. Our results should be further validated by prospective studies in multiple centers.

## Conclusions

We have generated a four-lncRNA prognostic risk score model consisting of *COMETT*, *EMX2OS*, *AC026462.3*, and *HAGLR*. The risk score was positively significantly correlated with the infiltration abundances of six immune cell types, including B cells, CD4 T cells, CD8 T cells, dendritic cells, macrophages, and neutrophils, suggesting the close and strong relationship with hypoxia microenvironment and tumor immune activity. The predictive nomograms integrating both the four-lncRNA signature and the conventional clinical–pathological risk factors were robust in predicting 5-, 7- and 10-year survival probabilities, which can help clinicians choose personalized treatment for ccRCC patients.

## Data Availability Statement

Publicly available datasets were analyzed in this study. This data can be found here: Publicly available datasets were analyzed in this study. This data can be found here: (https://portal.gdc.cancer.gov/).

## Ethics Statement

The studies involving human participants were reviewed and approved by The First Affiliated Hospital of Chongqing Medical University. The patients/participants provided their written informed consent to participate in this study.

## Author Contributions

HC contributed to conception and design of the study. GC organized the database. YP and XJ performed the statistical analysis. HC wrote the first draft of the manuscript. All authors contributed to the article and approved the submitted version.

## Funding

The authors declare that this study received funding from the Chongqing Science and Technology Commission (cstc2015shmszx120067). The funder was not involved in the study design, collection, analysis, interpretation of data, the writing of this article, or the decision to submit it for publication.

## Conflict of Interest

The authors declare that the research was conducted in the absence of any commercial or financial relationships that could be construed as a potential conflict of interest.

## Publisher’s Note

All claims expressed in this article are solely those of the authors and do not necessarily represent those of their affiliated organizations, or those of the publisher, the editors and the reviewers. Any product that may be evaluated in this article, or claim that may be made by its manufacturer, is not guaranteed or endorsed by the publisher.

## Supplementary Material

Supplementary Figure 1 Testing the performance of the signature in progression-free survival of ccRCC.

Supplementary Figure 2 Expression patterns of four lncRNAs between ccRCC and normal tissues. Figures were generated and downloaded from the online database GEPIA (http://gepia.cancer-pku.cn/index.html).

## Abbreviations

lncRNA, long non-coding; ccRCC, clear cell renal cell carcinoma; TIMER, Tumor IMmune Estimation Resource; TCGA KIRC, The Cancer Genome Atlas Kidney Renal Clear Cell Carcinoma; qRT-PCR, quantitative real-time PCR; adj. P-value, Adjusted P-value; FPKM, fragments per kilobase million; logFC, log (fold change); LASSO, Least Absolute Shrinkage and Selector Operation; ROC, receiver operating characteristic; GSEA, gene set enrichment analysis; AUC, area under curve. ESTIMATE, Estimation of STromal and Immune cells in MAlignant Tumors using expression data; *COMETT*, Cytosolic Oncogenic Antisense To MET Transcript; *EMX2OS*, EMX2 Opposite Strand/Antisense RNA; *HAGLR*, HOXD Antisense growth-associated long non-coding RNA; KM, Kaplan–Meier; *TGFBR2*, transforming growth factor beta receptor 2.
