# Supplementary figures and images for: Identification of a Four Hypoxia-Associated Long Non-Coding RNA Signature and Establishment of a Nomogram Predicting Prognosis of Clear Cell Renal Cell Carcinoma
Source: Front Oncol. 2021 Jul 27;11:713346. doi: 10.3389/fonc.2021.713346 (PMC8353455; doi:10.3389/fonc.2021.713346)

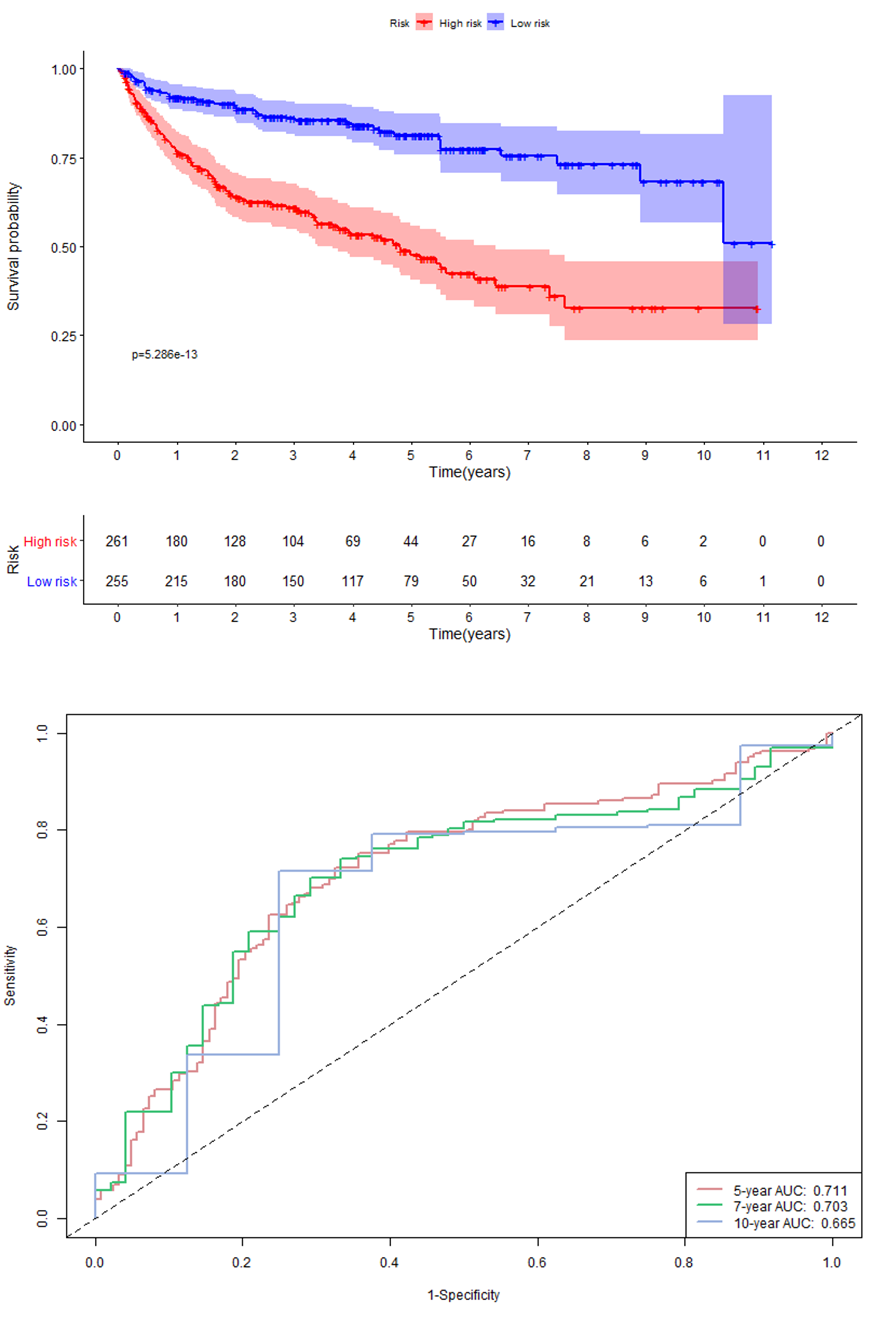

Supplement: Supplementary Figure 1 — Testing the performance of the signature in progression-free survival of ccRCC. [file Image_1.tif]

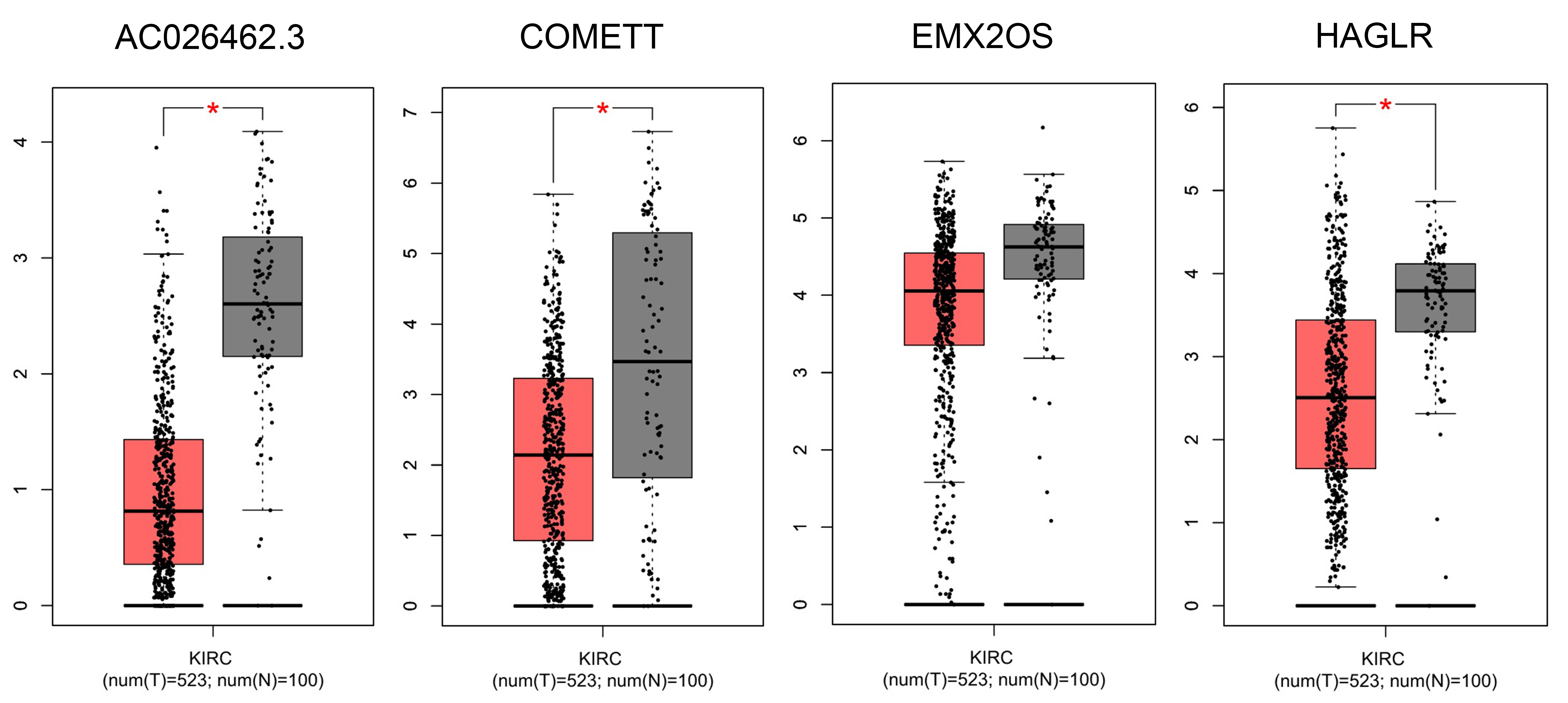

Supplement: Supplementary Figure 2 — Expression patterns of four lncRNAs between ccRCC and normal tissues. Figures were generated and downloaded from the online database GEPIA (http://gepia.cancer-pku.cn/index.html). [file Image_2.tif]
